# The efficacy of limited endoscopic sphincterotomy plus endoscopic papillary large balloon dilation for removal of large bile duct stones

**DOI:** 10.1186/s12876-019-1017-x

**Published:** 2019-06-18

**Authors:** Chung-Mou Kuo, Yi-Chun Chiu, Chih-Ming Liang, Cheng-Kun Wu, Lung-Sheng Lu, Wei-Chen Tai, Yuan-Hung Kuo, Keng-Liang Wu, Seng-Kee Chuah, Chung-Huang Kuo

**Affiliations:** Division of Hepato-Gastroenterology, Department of Internal Medicine, Kaohsiung Chang Gung Memorial Hospital and Chang Gung University College of Medicine, 123 Ta Pei Road, Niao-Sung Dist, 833 Kaohsiung, Taiwan

**Keywords:** Large bile duct stone extraction, Endoscopic sphincterotomy, Endoscopic papillary balloon dilation, Limited endoscopic sphincterotomy plus endoscopic papillary large balloon dilation, Complications

## Abstract

**Background:**

The removal of large bile duct stones (> 15 mm) by conventional endoscopic sphincterotomy (EST) and endoscopic papillary balloon dilation (EPBD) can be challenging, requiring mechanical lithotripsy (ML) in addition to EST or EPBD. The primary complication of ML is basket and stone impaction, which can lead to complications such as pancreatitis and cholangitis. The present study aims to investigate the efficacy of limited EST plus endoscopic papillary large balloon dilation (EST-EPLBD) for large bile duct stone extraction with an extent of cutting < 1/2 the length of the papillary mound.

**Methods:**

We enrolled 185 patients with ≥15 mm bile duct stones who received EST, EPLBD and limited EST-EPLBD treatment from January 1, 2010 to February 28, 2018, at Kaohsiung Chang Gung Memorial Hospital (Kaohsiung, Taiwan). All patients were categorized into three groups: EST group (*n* = 31), EPLBD group (*n* = 96), and limited EST-EPLBD group (*n* = 58). The primary outcome variables were the success rate of complete stone removal and complications.

**Results:**

The limited EST-EPLBD group exhibited a higher success rate of the first-session treatment compared with the EST and EPLBD groups (98.3% vs. 83.9% vs. 86.5%; *P* = 0.032) but required a longer procedure time (32 (12–61) min vs. 23.5 (17–68) min vs. 25.0 (14–60) min; *P* = 0.001). The need for ML during the procedure was 4 (12.9%) in the EST group, 10 (10.4%) in the EPLBD group and 2 (3.4%) in the limited EST-EPLBD group. Post-procedure bleeding in the EST group was more common than that in the limited EST-EPLBD group (9.7% vs. 0%; *P* = 0.038). Furthermore, dilated bile duct was the only risk factor for bile duct stone recurrence in the limited EST-EPLBD group.

**Conclusions:**

Limited EST-EPLBD exhibits a higher success rate but requires marginally longer procedure time for the first-session treatment. Furthermore, dilated bile duct is the only risk factor for bile duct stone recurrence in patients undergoing limited EST-EPLBD.

## Background

It is very troublesome when biliary tract disease such as bile duct stones are complicated with cholangitis, obstructive jaundice, and pancreatitis. Endoscopic retrograde cholangiopancreatography (ERCP) is the best option in order to remove bile duct stones. This skill for the removal of stones involves the initial common bile duct (CBD) cannulation, subsequent papilla opening broadening. It can be done by either endoscopic sphincterotomy (EST) or endoscopic papillary balloon dilation (EPBD). However, EST and EPBD can cause complications such as bleeding, perforation, cholangitis, and pancreatitis [1, 2]. In general, approximately 5–15% of bile duct stones failed to be detached with single technique of EST or EPBD, even in combination with the standard size balloon and basket extraction procedures [1, 3–7]. There were several reports emphasizing that the characteristics and locations of stones were associated with failure of bile duct stones extraction. These included the bigger size of stones of 15 mm or more, numerous stones, rigid stones, drum-shaped stones, stones above the bile duct stricture, the distal CBD narrowing, firmed and totally impacted bile duct stones, intrahepatic duct stones [1, 2, 4, 6–11]. It was well understood that EPBD had been applied to stones smaller than 10 mm only. Unlike EST, EPBD could not expand the duct orifice as wide as EST did [12]. The EST size needed to be adapted to the CBD and papilla size and which allowed approximately 80–90% of bile duct stones to be successfully extracted by EST, followed by retrieval balloons and baskets. Nevertheless, when EST was used alone, it might fail in larger stones extraction [2]. Whenever applicable, before the ERCP procedure, large bile duct stones removal might need the concomitant use of mechanical lithotripsy (ML) or intraductal electrohydraulic lithotripsy (EHL) or laser lithotripsy, or extracorporeal shock wave lithotripsy [13]. Most endoscopists used EST or EPBD combined with ML to remove large bile duct stones. However, we must keep in mind the possibility of procedure-related severe complications like the so-called “basket and stone impaction” which usually needs surgical interventions [2]. The main concern for ML was that the procedure needs more procedure time and is associated with increased risk of pancreatitis and cholangitis [2, 11]. To date, most studies have focused only on evaluating and comparing the efficacy and complications of EST alone and EST plus endoscopic papillary large balloon dilation (EPLBD) [3, 5, 6, 9, 14–19] or EPLBD alone and EPLBD plus EST or small or limited EST for the removal of large bile duct stones [1, 2, 10, 11, 20].

In addition, all these procedures might correlate with complications such as pancreatitis and bleeding. Some recent studies have reported the efficacy of EPLBD alone or combined with EST or small or limited EST, establishing it as a safe treatment for the removal of large bile duct stones [1–12, 14–22]. At our centre, we use EST, EPLBD, and limited EST plus endoscopic papillary large balloon dilation (EST-EPLBD) to remove large bile duct stones (≥15 mm). Theoretically, limited EST-EPLBD should be at least equally as effective as other techniques in stone extraction, with fewer complications. This study aims to investigate the efficacy and potential procedure-related complications of limited EST-EPLBD for large bile duct stone extraction.

## Methods

### Patients

In this retrospective study, we examined consecutive patients undergoing ERCP for large bile duct stones (≥15 mm) treatment. We enrolled 185 patients with large bile duct stones (≥15 mm) who received EST, EPLBD, or limited EST-EPLBD treatment between January 1, 2010 and February 28, 2018, at Kaohsiung Chang Gung Memorial Hospital (Kaohsiung, Taiwan). All patients were categorized into three groups: EST group (*n* = 31); EPLBD group (*n* = 96); and limited EST-EPLBD group (*n* = 58); Table 1 summarizes the characteristics and procedure findings of these three groups.

**Table 1:** Characterstics and procedure findings of three groups

	EST (*n* = 31)	EPLBD (*n* = 96)	Limited EST + EPLBD (*n* = 58)	*P*-value	Multiple comparisons
Gender (M/F)	18/13	49/47	26/32	0.481	
Age (years)	68 (45–88)	74 (37–99)	73.5 (55–98)	0.054	
Largest stone size (mm)	17 (15–27)	16.5 (15–26)	18 (15–35)	0.487	
Stone number	1: 14 (45.2%)	1: 40 (41.7%)	1: 28 (48.3%)	0.642	
	2: 8 (25.8%)	2: 19 (19.8)	2: 7 (12.1%)		
	3: 3 (9.7%)	3: 7 (7.3%)	3: 6 (10.3%)		
	> 3: 6 (19.4%)	> 3: 30 (31.3%)	> 3: 17 (29.3%)		
Largest CHD/CBD diameter(mm)	20 (11–27)	18 (12–35)	18 (11–40)	0.640	
Gallstones	15 (48.4%)	47 (49%)	22 (38%)	0.385	
Post-cholecystectomy	12(38.7%)	34 (35.4%)	27 (46.6%)	0.390	
Diverticulum	14 (45.2%)	47 (49%)	26 (44.8%)	0.861	
Procedure time of 1st session treatment (min)	23.5 (17–68)	25.0 (14–60)	32.0 (12–61)	0.001	EST = EPLBD <Limited EST + EPLBD

*EST* endoscopic sphincterotomy, *EPLBD* endoscopic papillary large balloon dilation, *CHD* common hepatic dust, *CBD* common bile duct

We defined a large bile duct stone as a stone ≥15 mm in the transverse diameter. The exclusion criteria were as follows: (1) segmental stricture or severe angulation of CBD or commom hepatic duct (CHD); (2) malignancy of CHD, CBD, pancreas and the ampulla Vater; (3) history of liver transplantation; (4) history of total or subtotal gastrectomy; (5) primary sclerosing cholangitis; (6) intrahepatic duct stones; (7) pregnancy; (8) refusal of written informed consent; and (9) uncorrected coagulopathy or bleeding tendency. When the patients were using anti-coagulants and with low risks of cardiovascular events for primary prevention, for instance aspirin, we stopped the medication 7-day before the procedure. On the other hand, we stopped clopidogrel, ticagrelor, and coumadin 5 days before ERCP for those patients who took the medications for secondary prevention [23, 24].

We used the methodology described in our previous publications to prepare patients for ERCP [25]. These preparations included intramuscular injection of 20-mg hyoscine-N-butybromide, 30–50 mg of meperidine (for only 1–2 min) and pharyngeal anesthesia with xylocaine spray. General anesthesia was performed for patients with poor tolerance and cooperation; patients were inducted by propofol, fentanyl/alfentanil and cis-atracurium. After successful intubation, we used inhalational anesthesia maintained by sevoflurane 1–3% or desflurane 3–8% for ERCP.

In our hospital, side-view endoscope (JF 260 V and TJF 240; Olympus, Tokyo, Japan) was available to perform ERCP. After the selective cannulation of CBD by using a cholangiography catheter (PR-113Q; Olympus) and 0.035-in. guidewire (Zebra Exchange Guidewire) was successfully achieved, a sphincterotome (ENDO-FLEX GmbH, Germany) was performed in all instances. We applied precut sphincterotomy in 26 patients (EST group, 10; limited EST-EPLBD group, 16) owing to difficult biliary cannulation. Three highly experienced endoscopists (the first, second, and corresponding authors who have an experience of > 2000 career ERCPs each, with an ongoing workload of > 200 ERCPs annually) performed all procedures using EST, EPLBD, and limited EST-EPLBD. In addition, diathermy was applied with the blended current (20 W cut; 20 W coagulation) in the ESG 100 System (Olympus). The EST was completed to its complete length by extending the incision up but not exceeding the major horizontal fold. When performing the limited EST-EPLBD, the extent of the cutting was less than 1/2 length of the papillary mound. A 0.035-in. guidewire (Zebra Exchange Guidewire; Microvasive Boston Scientific, Watertown, MA) was inserted into the bile duct through the catheter to conduct EPLBD after the bile duct cannulation by limited EST. After deeply inserting the guidewire into the bile duct, the catheter was removed with the guidewire left in place. Then, a large balloon-tipped catheter (5.5 cm × 12–20 mm CRE balloon; Microvasive Boston Scientific) was inserted over the guidewire so that the balloon was extended across the papilla. Next, the balloon was inflated to 12–20 mm in diameter with a saline solution to dilate the papilla at progressively increasing pressure of 3–8 atm for 2 min followed by 1 atm 1 min to prevent bleeding. After the dilation catheter removal, stones were extracted with a basket catheter or retrieval balloon. When the extraction of stones after EST or EPLBD was challenging, stones were crushed using endoscopic ML. When stones were not extracted completely, a plastic biliary stent was inserted, and the residual stones were removed after 3–7 days without repeating EST, EPLBD, or ML.

In current study, a prophylactic indomethacin suppository was routinely administered to all patients to decrease the risk of post-ERCP pancreatitis. When a balloon occlusion cholangiogram did not show the presence of any bile duct stone, the extraction of stones was considered successful.

### Definitions

We defined procedure-related complications as only adverse events associated with the procedure, including pancreatitis, bleeding, perforation, and cholangitis. Acute pancreatitis was defined as abdominal pain occurring within 24 h after the procedure in association with high serum amylase and lipase equivalent to at least three times the normal ranges and basal levels the day after ERCP [25]. We defined bleeding as any drop of > 15% in the haemoglobin level, any clinical sign of gastrointestinal bleeding (e.g., haematemesis and tarry stool), or the need for blood transfusion [25]. In addition, perforation was defined as the leakage of the contrast medium into the retroperitoneum or intraabdominal cavity during ERCP or the evidence of retroperitoneal-free air on abdominal plain radiography or computed tomography. Finally, cholangitis was defined as the presence of fever and/or chills, abdominal pain, jaundice, and leukocytosis.

### Statistical analysis

In this study, statistical analyses were performed using the statistical software package SPSS 22 for Windows (SPSS, Inc., Chicago, IL). All data for continuous variables are presented as the median and interquartile range (IQR), whereas percentages are used to present data for categorical variables. We used Pearson’s chi-square test and Fisher’s exact test to test for correlations between categorical variables. The Kruskal-Wallis test, multiple comparisons, and the nonparametric Mann–Whitney *U*-test were used to test for correlations between continuous variables. In addition, multivariate analysis was performed using logistic regression. We considered *P* < 0.05 as statistically significant.

## Results

We examined 185 patients (93 males and 92 female) with ≥15-mm large bile duct stones who underwent EST, EPLBD, and limited EST-EPLBD treatment. Table 1 summarizes the characteristics and procedure findings of the study cohort. We observed no significant differences among the three groups in sex, age, largest stone size, and stone number, dilation of the bile duct with largest diameter (CHD or CBD), gallbladder status, or periampullary diverticulum. The limited EST-EPLBD group exhibited a marginally longer procedure time of the first-session treatment [32.0 (12–61) min] compared with the EST group [23.5 (17–68) min] and the EPLBD group [25.0 (14–60) min; *P* = 0.001; Table 1]. Table 2 presents the therapeutic outcomes and complications of the three groups. The limited EST-EPLBD group exhibited similar outcomes in the overall successful stone removal (98.3%) compared with the EST (93.5%) and EPLBD groups (92.7%; *P* = 0.337). The limited EST-EPLBD group exhibited a higher success rate of the first-session treatment (98.3%) compared with the EST (83.9%) and EPLBD groups (86.5%; *P* = 0.032). In addition, 3 patients (10.3%) in the EST group and 6 patients (6.7%) in the EPLBD group received second-session treatment for bile duct stone removal. The EST group exhibited a higher post-procedure bleeding rate (9.7%) than the limited EST-EPLBD group (0%; *P* = 0.038). The need for ML was 4 patients (12.9%) in the EST group, 10 patients (10.4%) in the EPLBD group, and 2 patients (3.4%) in the limited EST-EPLBD group (12.9% vs. 10.4% vs. 3.4%; *P* = 0.215; Table 2). We observed no significant difference among the three groups in post-procedure pancreatitis (*P* = 0.852), perforation, cholangitis, or recurrent bile duct stones. Moreover, no procedure-related perforation or mortality was reported in the three groups. Notably, 4 patients (4.2%) in the EPLBD group and 5 patients (8.6%) in the limited EST-EPLBD group encountered recurrent bile duct stones during the follow-up period. Furthermore, the univariate and multivariate analysis revealed that the dilation of the bile duct with the largest diameter (CHD or CBD) was the only risk factor for bile duct stone recurrence in the limited EST-EPLBD group (*P* = 0.022; Table 3).

**Table 2:** Therapeutic outcomes and complications of three groups

	EST (*n* = 31)	EPLBD (*n* = 96)	Limited EST + EPLBD (*n* = 58)	*P*-value	Multiple comparisons
Overall success rate	29 (93.5%)	89 (92.7%)	57 (98.3%)	0.337	
Success rate of 1st session treatment	26 (83.9%)	83 (86.5%)	57 (98.3%)	0.032	EST = EPLBD<Limited EST + EPLBD
Mechanical lithotripsy	4 (12.9%)	10 (10.4%)	2 (3.4%)	0.215	
Pancreatitis	1 (3.2%)	2 (2.1%)	2 (3.4%)	0.852	
Bleeding	3 (9.7%)	4 (4.2%)	0	0.038	EST > Limited EST + EPLBD
Perforation	0	0	0	–	
Cholangitis	0	1 (1.0%)	0	> 0.99	
R Recurrent bile duct stone	0	4 (4.2%)	5 (8.6%)	0.369	

*EST* endoscopic sphincterotomy, *EPLBD* endoscopic papillary large balloon dilation

**Table 3:** Five patients with recurrent bile duct stone in limited EST plus EPLBD group

	Recurrence (*n* = 5)	No recurrence (*n* = 52)	*P*-value
Age (years)	84 (62–86)	72 (65.25–83.75)	0.682
Gender (M/F)	0/5	25/27	0.061
Gallstones	3	19	0.364
Post-cholecystectomy	2	24	> 0.99
Diverticulum	3	22	0.645
Stone number ≤ 3/> 3	5/0	35/17	0.157
Largest stone size (mm)	19.0 (17.0–27.5)	17.5 (15.0–20.0)	0.186
Procedure time (min)	41.0 (31.0–55.0)	31.5 (25.0–43.75)	0.127
Largest CHD/CBD diameter (mm)	2.60 (2.10–3.10)	1.80 (1.50–2.28)	0.022

*EST* endoscopic sphincterotomy, *EPLBD* endoscopic papillary large balloon dilation, *CHD* common hepatic dust, *CBD* common bile duct

## Discussion

The literature offers no clear definition of or any consensus statement regarding a large bile duct stone. Some studies suggested that a stone with a diameter equal to the CBD diameter is large or have used the term “difficult stone” when referring to large stone size. Most studies defined a stone > 10–15 mm in diameter as a “large stone” [2]. In most studies, the overall success rate for ≥10- or ≥ 12-mm bile duct stone removal was 74–100% in the EST group [2, 6, 9, 16, 17, 19], 89–100% in the EPLBD group [1, 2, 4, 10–12, 16, 20, 21], and 83–100% in the EPLBD + EST group [1, 2, 5, 6, 9, 10, 17, 19, 20]. In addition, the success rate of the first-session treatment for ≥10- or ≥ 12-mm bile duct stone removal was 81.4–85% in the EST group [9, 16, 17], 65–89% in the EPLBD group [2, 10, 12, 16, 20, 21], and 71–97% in the EPLBD + EST group [2, 7–10, 17]. In particular, large bile duct stones ≥15 mm yielded an overall success rate of 88.9–100% for stone removal in the EST group, 97.35–100% in the EPLBD group, and 94.4–100% in the EPLBD + EST group [1, 3, 4, 14, 15, 18]. The success rates of the first-session treatment for ≥15-mm bile duct stone removal were 55.6–87% in the EST group, 78–87.87% in the EPLBD group, and 83–88.2% in the EPLBD + EST group [3, 4, 14, 15, 18]. In this study, the overall success rate for ≥15-mm bile duct stone removal was 93.5% in the EST group, 92.7% in the EPLBD group, and 98.3% in the limited EST-EPLBD group. The success rate of the first-session treatment for ≥15 mm bile duct stone removal was 83.7% in the EST group, 86.5% in the EPLBD group, and 98.3% in the limited EST-EPLBD group. The limited EST-EPLBD group exhibited a higher success rate of the first-session treatment compared with the EST and EPLBD groups (*P* = 0.032; Table 2), which can be illustrated by the fact that limited EST-EPLBD can dilate both the duodenal papilla opening and the distal CBD simultaneously, facilitating large bile duct stone removal. In one patient, upon the partial extraction of stones, a plastic biliary stent was inserted, and the residual stones were removed after 3–7 days without repeating EST, EPLBD, or ML; this patient was considered one case in the analysis.

This study reported similar results on the need for ML and post-procedure complications for the removal of ≥15-mm bile duct stones compared with most studies [3, 4, 14, 15, 18]. Perhaps, the need for ML decreased in the limited EST-EPLBD group (3.4%) compared with the EST (12.9%) and EPLBD groups (10.4%), but this difference exhibited no statistical significance (*P* = 0.215; Table 2). In addition, no significant difference was noted in post-procedure pancreatitis (*P* = 0.852) or cholangitis (*P* > 0.99) among the three groups (Table 2). Of note, the risk of post-procedure pancreatitis did not increase in the limited EST-EPLBD group in this study, which could be because after limited EST, the force exerted by the dilating balloon is directed more towards the CBD than the pancreatic orifice. Reportedly, limited EST before EPLBD can decrease the risk of post-procedure pancreatitis [7, 8]. We observed that the post-procedure bleeding rate in the EST group (9.7%) was higher than that in the limited EST-EPLBD group (0%; *P* = 0.038). Perhaps, this difference could be attributed to the complete extent of the incision in the EST group and balloon dilation with compression of a possibly bleeding vessel after EST in the limited EST-EPLBD group. Hence, limited EST-EPLBD is recommended for the treatment of large bile duct stones in patients with an underlying coagulopathy or the need for anticoagulation following ERCP because of lower risk of bleeding than EST.

In some studies, the procedure time of the EPLBD + EST group was shorter than that of the EST group [5, 9, 15, 17]. However, the procedure time of the first-session treatment of the limited EST-EPLBD group was marginally prolonged compared with the EST and EPLBD groups (*P* = 0.001) in this study; the possible reasons were the dilation time of the CRE balloon and the removal of different sizes of large bile duct stones. In this study, the dilation time for the CRE balloon was 3 min compared with 15–30 s or 60 s in many previous studies [9, 15, 17]. Furthermore, the size of large bile duct stones was ≥15 mm in this study compared with ≥10 mm in most studies [9, 17].

In addition, 4 patients (4.2%) in the EPLBD group and 5 patients (8.6%) in the limited EST-EPLBD group encountered recurrent bile duct stones during the follow-up period. The recurrence rate (8.6%) of bile duct stones in our limited EST-EPLBD group corroborated other published studies (7.5–12.5%) [26, 27]. Furthermore, the multivariate analysis revealed that the dilation of the bile duct with the largest diameter (CHD or CBD) was the only statistically significant risk factor (*P* = 0.022), which corroborated the literature [26, 27].

This study has some limitations. First, this was a retrospective study conducted in a single centre with potential biases in the selection of patients and procedures. Thus, comprehensive prospective, randomized comparative studies are warranted to evaluate the differences among limited EST-EPLBD, EST, and EPLBD for large bile duct stone removal. Second, we enrolled consecutive patients undergoing ERCP for ≥15-mm bile duct stone treatment. Patients received EST, EPLBD, or limited EST-EPLBD treatment after comprehending the complications and efficacy of treatment and discussion with examiners. However, most procedure-related pancreatitis cases were mild for patients who received EPBD or EPLBD treatment at our hospital. Most patients were afraid of procedure-related duodenal bleeding and perforation at our hospital, and only a small number of patients received EST treatment. Third, we used a cholangiography catheter (PR-113Q) and 0.035-in. guidewire (Zebra Exchange Guidewire) for cannulation. We performed precut sphincterotomy in 26 patients (EST group, 10; limited EST-EPLBD group, 16) owing to difficult biliary cannulation. Although we attained 85.6% (159/185) successful cannulation in this study using a cholangiography catheter and 0.035-in. guidewire, the results could have been affected.

## Conclusions

This study suggests that limited EST-EPLBD could serve as an effective and safe treatment for the removal of ≥15-mm bile duct stones because of the higher success rate of the first-session treatment, with a similar complication rate and need for ML for the removal of ≥15-mm bile duct stones compared with EST and EPLBD. Limited EST-EPLBD offers the advantage of minimizing post-procedure bleeding despite needing marginally longer procedure time compared with EST and EPLBD. Furthermore, the multivariate analysis revealed that the dilation of the bile duct with the largest diameter (CHD or CBD) was the only risk factor for bile duct stone recurrence in the limited EST-EPLBD group.

## Data Availability

The datasets generated during the current study are available in the manuscript.

## Abbreviations

CBD: Common bile duct
CHD: Common hepatic duct
CRE: Controlled radial expansion
EHL: Electrohydraulic lithotripsy
EPBD: Endoscopic papillary balloon dilation
ERCP: Endoscopic retrograde cholangiopancreatography
EST: Endoscopic sphincterotomy
EST-EPLBD: Limited EST plus endoscopic papillary large balloon dilation
IQR: Interquartile range
ML: Mechanical lithotripsy
